# Examining facilitative services for entry into substance use disorder treatment: A cluster analysis of treatment facilities

**DOI:** 10.1371/journal.pone.0304094

**Published:** 2024-05-23

**Authors:** Emmanuel O. Amoako, Lisa D. Zerden, Tamera D. Hughes, Alex K. Gertner, Joseph Williams, C. Micha Belden, Orrin D. Ware

**Affiliations:** 1 School of Social Work, University of North Carolina at Chapel Hill, Chapel Hill, NC, United States of America; 2 Cecil G. Sheps Center for Health Services Research, University of North Carolina at Chapel Hill, Chapel Hill, NC, United States of America; 3 Eshelman School of Pharmacy, University of North Carolina at Chapel Hill, Chapel Hill, NC, United States of America; 4 University of North Carolina Hospitals, Chapel Hill, NC, United States of America; 5 School of Medicine, University of North Carolina at Chapel Hill, Chapel Hill, NC, United States of America; 6 Mountain Area Health Education Center, Asheville, NC, United States of America; University of Jyvaskyla, FINLAND

## Abstract

**Objectives:**

We examined services to facilitate access to entering substance use disorder (SUD) treatment among a national sample of SUD treatment facilities.

**Methods:**

We analyzed data from the National Survey of Substance Abuse Treatment Services (N-SSATS) 2020. Facilities were included in the sample based on criteria such as SUD treatment provision and being in the U.S. Cluster analysis was conducted using variables including ownership, levels of care, and whether facilities provide services or accept payment options aimed at reducing treatment barriers. National and state-level data on the percentage of facilities in each cluster were presented.

**Results:**

Among N = 15,788 SUD treatment facilities four distinct clusters were identified: Cluster 1 consisted of for-profit and government outpatient facilities with high proportions of services to reduce barriers (22.2%). Cluster 2, comprised of non-profit outpatient facilities, offered the most comprehensive array of services to minimize barriers to treatment among all four clusters (25.2%). Cluster 3 included facilities with diverse ownership and care levels and provided a moderate to high degree of services aimed at reducing entry barriers to treatment (26.0%). Cluster 4 was primarily for-profit outpatient facilities with a low proportion of these services (26.6%).

**Conclusions:**

This study revealed facility-level groupings with different services to reduce barriers to SUD treatment across various clusters of SUD treatment facilities. While some facilities offered extensive services, others provided fewer. Differences in cluster distributions point to possible facilitators to treatment access for some persons seeking admission to specific treatment facilities. Efforts should be made to ensure that individuals seeking SUD treatment can access these services, and facilities should be adequately equipped to meet their diverse needs.

## Introduction

Treatment has been shown to reduce morbidity and mortality associated with having a substance use disorder (SUD) [1–3]. Despite the benefits of SUD treatment, many individuals face barriers to accessing treatment, furthering their potential exposure to the harmful outcomes related to SUDs. Examples of barriers that individuals may face in accessing SUD treatment include geographic access (e.g., limited or no available treatment programs locally, which could vary by region, state, county, or district), fears about treatment, criminalization of illicit substance use, costs associated with treatment, lack of insurance, transportation, and no current openings in treatment programs [4–6]. National and state-level studies investigating the collection of services offered by substance use treatment organizations that may improve treatment access are limited [7–9]. To combat structural barriers to treatment (e.g. costs of treatment, lack of transportation to treatment) and improve treatment accessibility, some SUD treatment facilities provide services that facilitate or promote entry into treatment [4, 10]. Facilitative factors to entering treatment may include flexible payment options, housing support, transportation assistance, or assistance obtaining social services. However, little is known about the distribution of SUD treatment providers that offer these different ancillary services in the U.S. Therefore, an examination of the groupings of facilitators provided by these treatment facilities is needed.

Socioeconomic factors often hamper one’s access to treatment. A prominent barrier to accessing SUD treatment is the inability to pay for these services, including insurance coverage gaps [4, 11–13]. The Patient Protection and Affordable Care Act (ACA) boosted insurance coverage rates among people with SUD [14]. However, a study that examined SUD treatment access pre-ACA (2009–2013) and post-ACA (2015–2019) did not find a significant increase in treatment entry among adults who have a SUD with a lower socioeconomic status [5]. Instead, the authors found that while some barriers to accessing treatment increased during the analytical period (e.g., lack of knowledge of available services, perceived stigma, and difficulties accessing appropriate treatment options), there was no significant reduction in insurance-related treatment barriers, highlighting the importance of considering multiple factors to increase SUD treatment access [4, 5, 15].

Individuals with lower socioeconomic status may encounter difficulties pertaining to finances, housing, and social assistance [12]. These factors may intersect and compound, further hindering the capacity of persons to fully participate in the SUD treatment process. Interventions provided by SUD treatment facilities that address housing instability, access to transportation, and financial challenges experienced by persons needing SUD treatment services may enhance treatment access, engagement, and beneficial post-treatment outcomes [4, 15, 16]. Alongside these needed ancillary services, educational and outreach efforts to increase the knowledge of what characterizes a SUD and how to access treatment are necessary. Lack of motivation for seeking treatment, not recognizing the characteristics of a SUD, or being unfamiliar with the SUD treatment landscape are other potential barriers to treatment [17]. Education and outreach efforts provided by SUD facilities could increase familiarity and knowledge of SUD treatment, and reduce individual and community-level barriers, such as SUD stigma [4].

Understanding the nationwide availability of services provided by SUD treatment facilities as potential facilitators to entering treatment is imperative. This study provides an opportunity to identify current strengths, service gaps, and accessibility issues related to SUD treatment entry. Specifically, this current study examined services to reduce barriers to entering treatment among a national sample of SUD treatment facilities. We focused on facilitators in three primary domains, (1) care coordination services, (2) flexible payment options, and (3) social support. Facilities were further clustered to examine similar groupings of facilities based on their ownership (e.g. for-profit, government, and non-profit), levels of care, and services to reduce barriers to entering treatment.

## Materials and methods

### Data source

This study used the National Survey of Substance Abuse Treatment Services (N-SSATS) 2020, a publicly available dataset provided by the Substance Abuse and Mental Health Services Administration (SAMHSA), as a census of all known SUD treatment facilities in the U.S. during 2020 [18]. Representatives of treatment facilities can complete a SAMHSA survey online, by mail, or telephonically to describe their facility structure and the services that they provide [18]. In 2020, approximately 88% of SUD treatment facilities that were eligible (some facilities were determined to be ineligible because they closed) to complete the survey were included in the dataset [18]. The N-SSATS 2020 dataset contains data from 16,066 SUD treatment facilities in the US.

### Inclusion and exclusion criteria

The following inclusion criteria were applied to select the final analytic sample, 1) SUD treatment is provided (Variable: TREATMT; some facilities may only provide screening or referrals), and 2) based in the U.S. (Variable: STATE; not U.S. territories). Based on these criteria, 278 facilities were excluded, resulting in a final analytic sample of 15,788 SUD treatment facilities.

### Measures

Variables included in this study were all captured from N-SSATS 2020 and include (a) flexible payment option, (b) level of care, (c) ownership, (d) care coordination service, (e) social support service, and (f) telehealth.

#### Flexible payment option

The flexible payment option variable was created by merging five binary Yes/No variables in the dataset. These variables are (1) “FEESCALE” which indicates if the facility uses a sliding fee scale, (2) “PAYASST” which indicates if the facility has treatment at no or minimal charge for individuals who cannot afford to pay, (3) “REVCHK3” which indicates if the facility offers free treatment for everyone, (4) “REVCHK5” which indicates if the facility accepts Medicaid, and (5) “REVCHK8” which indicates if the facility accepts Medicare [18]. These variables were merged to identify if the facility had a flexible payment option. If a facility selected “Yes” to any of the five variables, they were identified as having a flexible payment option. Facilities that did not select Yes to any of the five variables were identified as not having a flexible payment option. The number of flexible payment options was also summed for each facility, with a potential range of 0 to 5.

#### Level of care

Level of care described if the facility offered three specific levels of care, (1) hospital inpatient, (2) non-hospital residential, and (3) outpatient. The hospital inpatient variable was created from “CTYPE4” in the N-SSATS 2020 dataset which is a binary Yes/No variable that indicates if the facility has any hospital inpatient SUD treatment [18]. The non-hospital residential variable was created from “CTYPE7” in the N-SSATS 2020 dataset, a binary Yes/No variable that indicates if the facility has non-hospital residential SUD treatment [18]. The outpatient variable was created from “CTYPE1” in the N-SSATS 2020 dataset, a binary Yes/No variable that indicates if the facility has any outpatient SUD treatment [18].

#### Ownership

The ownership variable was created from “OWNERSHP” in the N-SSATS 2020 dataset, a categorical variable that indicates the facility’s ownership designation [18]. Six values for this variable included (a) federal government, (b) local, county, or community government, (c) private-for-profit organization, (d) private non-profit organization, (e) state government, and (f) tribal government [18]. For this study, values were recoded into three categories, (1) for-profit, (2) government, and (3) non-profit.

#### Care coordination service

The care coordination service variable was created by merging three binary Yes/No variables in the dataset. These three variables are (1) “SRVC59” which indicates if the facility offers transportation assistance to SUD treatment, (2) “SRVC91” which indicates if the facility engages in community outreach for persons who may need SUD treatment, and (3) “SRVC93” which indicates if the facility offers services for persons when an immediate admission is not possible [18]. These variables were merged to identify if the facility had a care coordination service. If a facility selected Yes to any of the three variables, they were identified as having care coordination services. Facilities that did not select Yes to any of the three variables were identified as not having care coordination services. The number of care coordination services was also summed for each facility with a potential range of 0 to 3.

#### Social support service

The social support service variable was created by merging three binary Yes/No variables in the dataset: (1) “SRVC36” which indicates if the facility offers assistance with obtaining social services, (2) “SRVC38” which indicates if the facility offers employment counseling or training, and (3) “SRVC38” which indicates if the facility offers housing location services [18]. These variables were merged to identify if the facility had social support services. Facilities that did not select Yes to any of these three variables were identified as not having a social support service. The number of social support services was also summed for each facility with a potential range of 0 to 3.

#### Telehealth

The telehealth variable was created from “TELEMED” in the N-SSATS 2020 dataset, a binary Yes/No variable that indicates if the facility frequently uses telemedicine/telehealth therapy [18].

### Data analysis

Statistical analyses were conducted using IBM SPSS Statistics 28.0 [19]. Descriptive statistics were used to examine facilities. A Two-step cluster analysis with log-likelihood distance measure with Schwarz’s Bayesian Criterion (BIC) was utilized because it is a good exploratory tool to assist with identifying similar groups. Further, the BIC is used to identify which model is the best fit. The cluster analysis was used to identify facilities that are similar based on their ownership, levels of care, and whether they provide services or accept payment options that reduce barriers to entering treatment. Variables included in the cluster analysis are: (1) flexible payment option, (2) hospital inpatient, (3) non-hospital residential, (4) outpatient, (5) ownership, (6) care coordination service, and (7) social support service. Cluster analysis was selected as it is a good exploratory tool to assist with identifying similar groups within a dataset or sample. The average silhouette was examined to determine the quality of the clusters. Telehealth was not included in the cluster analysis as an a priori decision since hospital inpatient and non-hospital residential treatment service levels were included in the model. Descriptive statistics were used to identify the characteristics of facilities in each cluster. Ethical review was conducted by the University of North Carolina at Chapel Hill which applied the designation of not human subjects research.

## Results

### Sample characteristics

Descriptive data about the sample is provided in Table 1. Most of the facilities offered an outpatient level of care at 83.0% (*n* = 13,104). A slight majority of facilities frequently used telemedicine/telehealth at 59.2% (*n* = 9,349). Regarding ownership, the highest percentage of facilities were non-profit at 49.6% (*n* = 7,831), followed by for-profit at 41.0% (*n* = 6,471).

**Table 1:** Descriptive statistics of the full analytic sample and the four clusters.

	Entire Analytic Sample N = 15,788	Cluster 1: For-Profit and Government Outpatient Facilities with a High Proportion of Services to Reduce Barriers to Treatment n = 3,502 (22.2%)	Cluster 2: Non-Profit Outpatient Facilities with a High Proportion of Services to Reduce Barriers to Treatment n = 3,972 (25.2%)	Cluster 3: Primarily Non-Profit then For-Profit Mixed Levels of Care with a Medium to High Proportion of Services to Reduce Barriers to Treatment n = 4,107 (26.0%)	Cluster 4: Primarily For-Profit then Non-Profit Outpatient Facilities with a Low Proportion of Services to Reduce Barriers to Treatment n = 4,207 (26.6%)
**Ownership (Cluster Row %; Column %)** ^**1**^					
For-Profit	6,471 (41.0%)	2,491 (38.5%; 71.1%)	0 (0.0%; 0.0%)	1,290 (19.9%; 31.4%)	2,690 (41.6%; 63.9%)
Government	1,486 (9.4%)	1,011 (68.0%; 28.9%)	0 (0.0%; 0.0%)	349 (23.5%; 8.5%)	126 (8.5%; 3.0%)
Non-Profit	7,831 (49.6%)	0 (0.0%; 0.0%)	3,972 (50.7%; 100.0%)	2,468 (31.5%; 60.1%)	1,391 (17.8%; 33.1%)
**Level of Care (Cluster Row %; Column %)** ^**1**^					
Hospital Inpatient	759 (4.8%)	0 (0.0%; 0.0%)	0 (0.0%; 0.0%)	759 (100.0%; 18.5%)	0 (0.0%; 0.0%)
Non-Hospital Residential	3,685 (23.3%)	0 (0.0%; 0.0%)	0 (0.0%; 0.0%)	3,685 (100.0%; 89.7%)	0 (0.0%; 0.0%)
Outpatient	13,104 (83.0%)	3,502 (26.7%; 100.0%)	3,972 (30.3%; 100.0%)	1,423 (10.9%; 34.6%)	4,207 (32.1%; 100.0%)
**Has Care Coordination Service (Cluster Row %; Column %)** ^**1**^					
Yes	13,006 (82.4%)	3,448 (26.5%; 98.5%)	3,972 (30.5%; 100.0%)	3,458 (26.6%; 84.2%)	2,128 (16.4%; 50.6%)
No	2,782 (17.6%)	54 (1.9%; 1.5%)	0 (0.0%; 0.0%)	649 (23.3%; 15.8%)	2,079 (74.4%; 49.4%)
**Has a Flexible Payment Option (Cluster Row %; Column %)** ^**1**^					
Yes	13,818 (87.5%)	3,502 (25.3% 100.0%)	3,972 (28.7%; 100.0%)	3,327 (24.1%; 81.0%)	3,017 (21.8%; 71.7%)
No	1,970 (12.5%)	0 (0.0%; 0.0%)	0 (0.0%; 0.0%)	780 (39.6%; 19.0%)	1,190 (60.4%; 28.3%)
**Has a Social Support Service (Cluster Row %; Column %)** ^**1**^					
Yes	12,380 (78.4%)	3,388 (27.4%; 96.7%)	3,972 (32.1%; 100.0%)	3,729 (30.1%; 90.8%)	1,291 (10.4%; 30.7%)
No	3,408 (21.6%)	114 (3.3%; 3.3%)	0 (0.0%; 0.0%)	378 (11.1%; 9.2%)	2,916 (85.6%; 69.3%)
**Average Barrier Reduction Service (*Mean*; Standard Deviation)**					
Care Coordination Service^2^	1.6 (1.0)	2.0 (0.8)	2.2 (0.8)	1.7 (1.0)	0.8 (0.9)
Flexible Payment Option^3^	2.2 (1.3)	2.4 (1.0)	3.0 (0.9)	1.8 (1.2)	1.5 (1.3)
Social Support Service^2^	1.8 (1.2)	2.2 (0.9)	2.3 (0.8)	2.2 (1.0)	0.6 (1.0)
**Care Coordination Service (Column %)** ^**4**^					
Community Outreach	10,495 (66.5%)	3,001 (85.7%)	3,436 (86.5%)	2,500 (60.9%)	1,558 (37.0%)
Interim Services^5^	7,962 (50.4%)	2,257 (64.4%)	2,849 (71.7%)	1,654 (40.3%)	1,202 (28.6%)
Transportation	7,488 (47.4%)	1,806 (51.6%)	2,479 (62.4%)	2,684 (65.4%)	519 (12.3%)
**Flexible Payment Option Services (Column %)** ^**4**^					
Feescale	9,085 (57.5%)	2,161 (61.7%)	3,286 (82.7%)	1,853 (45.1%)	1,785 (42.4%)
Free Treatment^6^	380 (2.4%)	61 (1.7%)	117 (2.9%)	169 (4.1%)	33 (0.8%)
Medicaid	11,199 (70.9%)	2,900 (82.8%)	3,692 (93.0%)	2,377 (57.9%)	2,230 (53.0%)
Medicare	6,644 (42.1%)	1,801 (51.4%)	2,294 (57.8%)	1,089 (26.7%)	1,451 (34.5%)
Pay Assistance	6,872 (43.5%)	1,539 (43.9%)	2,675 (67.3%)	1,782 (43.4%)	876 (20.8%)
**Social Support Services (Column %)** ^**4**^					
Employment Counseling or Training	7,062 (44.7%)	1,852 (52.9%)	2,228 (56.1%)	2,381 (58.0%)	601 (14.3%)
Housing Location	10,061 (63.7%)	2,731 (78.0%)	3,157 (79.5%)	3,304 (80.4%)	869 (20.7%)
Assistance with Social Services	10,949 (69.4%)	3,033 (86.6%)	3,678 (92.6%)	3,222 (78.5%)	1,016 (24.2%)
**Facility Frequently Uses Telemedicine/Telehealth**	9,349 (59.2%)	2,376 (67.8%)	2,747 (69.2%)	1,977 (48.1%)	2,249 (53.5%)

^1^Row and column percentages were presented to show proportions between and within variables added to the cluster analysis.

^2^Possible Values 0 to 3.

^3^Possible Values 0 to 5.

^4^Seperate variables were grouped under these categories, therefore the specific services were not mutually exclusive and total percentages may be over 100.0%.

^5^Interim services when admission to treatment is not currently available.

^6^Treatment is free for everyone.

### Services to reduce barriers to treatment outcomes

Among the full sample, most facilities had at least one care coordination service (*n* = 13,006, 82.4%), one flexible payment option (*n* = 13,818, 87.5%), and one social support service (*n* = 12,380, 78.4%). The cluster analysis indicated a four-cluster solution with an average silhouette measure of cohesion and separation of 0.5, indicating good clustering.

The four clusters were conceptualized as the following, (1) Cluster 1: For-Profit and Government Outpatient Facilities with a High Proportion of Services to Reduce Barriers to Treatment (*n* = 3,502; 22.2%), (2) Cluster 2: Non-Profit Outpatient Facilities with a High Proportion of Services to Reduce Barriers to Treatment (*n* = 3,972; 25.2%), (3) Cluster 3: Primarily Non-Profit then For-Profit Mixed Levels of Care with a Medium to High Proportion of Services to Reduce Barriers to Treatment (*n* = 4,107; 26.0%), and (4) Cluster 4: Primarily For-Profit then Non-Profit Outpatient Facilities with a Low Proportion of Services to Reduce Barriers to Treatment (*n* = 4,207; 26.6%). Table 1 offers detailed characteristics of the four clusters.

The secondary cluster model with telehealth added as another variable identified three clusters but did not yield an adequate average silhouette at 0.3. Therefore, this secondary model was not interpreted further since it was suboptimal to the primary four-cluster solution.

### Cluster 1

The first cluster included for-profit and government outpatient facilities with flexible payment options and almost all having care coordination services and social services. This cluster accounted for the smallest grouping of approximately 22.2% across the nationwide sample of facilities. Cluster 1 provided the most services to reduce barriers to entering treatment for those receiving outpatient services from for-profit or government-based facilities. Further, facilities in this cluster had, on average, at least 2 care coordination services, 2 flexible payment options, and 2 social support services. Therefore, individuals entering treatment or needing care coordination services in facilities found in Cluster 1 may have fewer barriers to accessing SUD treatment.

### Cluster 2

Cluster 2 included non-profit outpatient facilities with care coordination services, flexible payment options, and social support services. This Cluster accounted for the second smallest grouping of facilities nationally at 25.2% and included facilities with an average of 2 care coordination services, 3 flexible payment options, and 2 social support services. Cluster 2 provided the most services to reduce barriers to entering treatment across all four clusters. However, only non-profit outpatient facilities were included in Cluster 2, whereas Cluster 1 provided the most accessibility for individuals receiving treatment from for-profit/government outpatient facilities.

### Cluster 3

The third Cluster included facilities with all three ownership types, all three levels of care, and medium to high services to reduce barriers to entering treatment. Although this Cluster had services that facilitated access to treatment ranging from approximately 81% to 91%, these facilities had a lower proportion of these services compared to Clusters 1 and 2, prompting the identification of a “medium to high proportion of services to reduce barriers.”

### Cluster 4

The fourth and final cluster was the largest group at 26.6% and included facilities with all three ownership types with outpatient treatment and a low proportion of services to reduce barriers to treatment. Cluster 4 represents outpatient services that provided the lowest proportion of services to reduce barriers to treatment.

### State level data

Table 2 shows the state-level percentages of facilities in each cluster and the status of Medicaid Expansion in each state [20]. Connecticut had the lowest percentage (5.8%) of facilities, and Idaho had the highest percentage (67.3%) in Cluster 1. Louisiana had the lowest percentage (4.5%) of facilities, and Vermont had the highest percentage (58.8%) in Cluster 2. Regarding Cluster 3, Vermont had the lowest percentage (11.8%), and California had the highest (45.3%). Vermont had the lowest percentage (7.8%) of facilities whereas New Hampshire had the highest percentage (43.1%) in Cluster 4.

**Table 2:** Region and state level proportions of the four clusters and medicaid adopted and implementation status by January 1, 2020.

**Region**		**Cluster 1** ^**1**^	**Cluster 2** ^**2**^	**Cluster 3** ^**3**^	**Cluster 4** ^**4**^
Northeast		17.4%	32.0%	27.3%	23.3%
Midwest		18.1%	27.9%	21.3%	32.6%
South		25.7%	19.5%	25.5%	29.4%
West		25.7%	19.5%	25.5%	29.4%
**State**	**Medicaid Expansion**	**Cluster 1** ^**1**^	**Cluster 2** ^**2**^	**Cluster 3** ^**3**^	**Cluster 4** ^**4**^
AK	Yes	29.8%	33.7%	25.0%	11.5%
AL	No	21.1%	28.6%	27.2%	23.1%
AR	Yes	14.4%	45.5%	18.0%	22.2%
AZ	Yes	25.3%	29.8%	24.8%	20.1%
CA	Yes	21.4%	17.3%	45.3%	16.0%
CO	Yes	18.0%	27.5%	13.9%	40.6%
CT	Yes	5.8%	47.6%	24.8%	21.8%
DC	Yes	14.3%	50.0%	21.4%	14.3%
DE	Yes	18.4%	34.7%	20.4%	26.5%
FL	No	15.4%	16.0%	32.9%	35.7%
GA	No	34.2%	9.6%	25.5%	30.7%
HI	Yes	11.2%	43.5%	14.3%	31.1%
IA	Yes	10.8%	37.8%	17.3%	34.1%
ID	Yes	67.3%	6.4%	12.7%	13.6%
IL	Yes	11.9%	32.9%	13.2%	42.1%
IN	Yes	13.3%	30.1%	19.0%	37.6%
KS	No	24.0%	31.1%	17.4%	27.5%
KY	Yes	22.1%	26.1%	21.2%	30.7%
LA	Yes	39.4%	4.5%	38.1%	18.1%
MA	Yes	10.6%	23.9%	36.8%	28.7%
MD	Yes	39.6%	14.0%	21.0%	25.4%
ME	Yes	20.1%	25.8%	11.9%	42.3%
MI	Yes	21.9%	23.0%	20.6%	34.5%
MN	Yes	27.2%	11.1%	30.2%	31.5%
MO	No	6.9%	47.8%	23.9%	21.4%
MS	No	13.6%	20.4%	42.7%	23.3%
MT	Yes	26.4%	27.9%	14.0%	31.8%
NC	No	31.3%	11.9%	17.2%	39.6%
ND	Yes	21.2%	5.9%	34.1%	38.8%
NE	No	16.4%	14.8%	29.5%	39.3%
NH	Yes	13.8%	27.5%	15.6%	43.1%
NJ	Yes	26.4%	26.4%	14.5%	32.7%
NM	Yes	34.2%	33.5%	12.4%	19.9%
NV	Yes	25.0%	29.6%	30.6%	14.8%
NY	Yes	16.3%	37.6%	32.9%	13.2%
OH	Yes	21.3%	36.9%	23.4%	18.4%
OK	No	29.4%	34.5%	25.3%	10.8%
OR	Yes	32.1%	36.6%	16.0%	15.2%
PA	Yes	21.6%	28.6%	29.0%	20.8%
RI	Yes	19.4%	30.6%	32.3%	17.7%
SC	No	35.0%	17.9%	26.0%	21.1%
SD	No	15.5%	24.1%	34.5%	25.9%
TN	No	18.3%	26.4%	27.3%	28.0%
TX	No	18.9%	20.9%	29.2%	31.0%
UT	Yes	27.1%	9.8%	34.1%	29.0%
VA	Yes	45.6%	6.0%	18.1%	30.2%
VT	Yes	21.6%	58.8%	11.8%	7.8%
WA	Yes	31.0%	33.9%	14.6%	20.4%
WI	No	27.7%	10.3%	21.2%	40.8%
WV	Yes	8.8%	37.6%	30.4%	23.2%
WY	No	11.7%	48.3%	31.7%	8.3%

The proportions are row percentages for each region and state.

Some percentages may not equal 100.0% due to rounding.

Counts are not provided to avoid identifying individual level treatment facilities

^1^Cluster 1: For-Profit and Government Outpatient Facilities with a High Proportion of Services to Reduce Barriers to Treatment

^2^Cluster 2: Non-Profit Outpatient Facilities with a High Proportion of Services to Reduce Barriers to Treatment

^3^Cluster 3: Primarily Non-Profit then For-Profit Mixed Levels of Care with a Medium to High Proportion of Services to Reduce Barriers to Treatment

^4^Cluster 4: Primarily For-Profit then Non-Profit Outpatient Facilities with a Low Proportion of Services to Reduce Barriers to Treatment

## Discussion

This paper presents four clusters from SUD treatment facilities by ownership, levels of care provided, and services that serve as facilitators to accessing treatment. The identified clusters were described as (1) Cluster 1: For-Profit and Government Outpatient Facilities with a High Proportion of Services to Reduce Barriers to Treatment, (2) Cluster 2: Non-Profit Outpatient Facilities with a High Proportion of Services to Reduce Barriers to Treatment, (3) Cluster 3: Primarily Non-Profit then For-Profit Mixed Levels of Care with a Medium to High Proportion of Services to Reduce Barriers to Treatment, and (4) Cluster 4: Primarily For-Profit then Non-Profit Outpatient Facilities with a Low Proportion of Services to Reduce Barriers to Treatment.

Considering the clusters that were identified, a differential in treatment access could be present if the types of services a SUD treatment facility offers do not match the barriers an individual needing treatment faces. Financial facilitators to accessing SUD treatment across facilities in all clusters ranged from 53.0% - 93.0% of facilities accepting Medicaid and between 42.4% - 82.7% of facilities offering a sliding fee scale. Sliding price scales based on income is a practical tactic that enables individuals to pay for care according to their financial capability [15]. Financial assistance is often needed as individuals with a SUD may be financially burdened by fines, fees, and legal charges [21].

While financial barriers to treatment can be a restrictive reason for not accessing care, for those in need of treatment, it is important to assess and intervene upon multiple factors that can increase treatment access broadly [4, 5, 15]. For example, treatment facilities offering services that assist individuals with employment training, housing support, and social services could reduce prominent individual-level barriers [22, 23] and promote a more secure foundation for recovery and increased general well-being. Treatment facilities offering assistance with obtaining social services like Low Income Home Energy Assistance (LIHEAP), Medicaid enrollment, Temporary Assistance for Needy Families (TANF), and Supplemental Nutrition Assistance Program (SNAP) are incredibly important to support vulnerable populations [24]. Also, assisting persons with applying for and obtaining Medicaid further reduces barriers to accessing needed healthcare. Not addressing the multi-morbidities associated with social, health, and economic needs of individuals with SUD, may cause individuals to delay treatment entry. Addressing basic needs related to employment, financial stability, housing, access to food, and healthcare coverage are all important and fundamental aspects associated with one’s ability to engage in SUD treatment and recovery [4, 5, 15].

In addition, this study looked at crucial care coordination services such as community outreach, interim services prior to receiving SUD treatment, and transportation to treatment. Community outreach services provide the potential for individuals, especially difficult-to-reach populations, to become familiar with available treatment options [25, 26]. Beyond the familiarity of treatment providers, getting to treatment is important. Not all persons have reliable methods of transportation; therefore, SUD treatment providers that offer transportation services to treatment increase access [27, 28]. Furthermore, SUD treatment providers may be at capacity for the number of persons to whom they can provide treatment. Waitlists for treatment admission also serve as barriers to treatment entry [29–31]. However, when SUD treatment facilities provide interim services for persons whom they cannot immediately accept, treatment facilities may reduce the risk of harmful outcomes (e.g., the risk of an accidental overdose). While it would be a gold standard for all facilities to offer an exhaustive list of services that are facilitators of SUD treatment access, this reality is hampered by a lack of staff and resources in different treatment facilities [4].

Findings from this study must be considered alongside study limitations. The N-SSATS dataset contains data from 88% of all treatment facilities, however, we are unable to generalize this study’s results to the remaining 12% of facilities. Another limitation is that this study does not include an exhaustive list of services that could be provided to reduce all barriers to SUD treatment nor does this analysis determine how the workforce at these facilities is appropriately staffed to meet all needs. Other barriers to treatment access include SUD and behavioral health workforce shortages [32], the distance from treatment facilities, rurality, and geographic location [33, 34], needed legal services, especially for persons with criminal justice involvement [35], stigma [4], availability of medications to treat SUD [36], and a lack of culturally appropriate care. While state-level Medicaid expansion status was examined in this paper descriptively, it was not included in the cluster analysis. However, the decision to not include Medicaid expansion as a cluster variable was twofold (1.) this study focused specifically on descriptives of treatment facilities, and (2.) thirty-two states and Washington, D.C. adopted and implemented Medicaid expansion by January 1, 2020, which would likely result in a Medicaid expansion variable driving the clusters. Further, since this study is cross-sectional, we are unable to examine the impact of any federal or state-level policies or why facility clusters may be different across each of the 50 states and Washington, D.C. Treatment facilities offering specific services could be due to state, county, or district level policies that are not examined in this current paper. Another limitation is this study did not examine the availability of facilities to admit new patients or the current number of patients receiving treatment. Future studies may examine these facilitative services alongside the admissions capacity and census. Also, the self-report nature of the N-SSATS may increase the risk of biased responses. Another limitation is that while the N-SSATS data are provided at the state level to avoid identifying facilities, the data does not allow for examination based on more specific geographic designations (e.g., suburban, rural, or urban) which impact where facilities are located and how accessible they can be based on physical location alone. For future replication purposes, it should also be noted that the year 2020 was the final year of N-SSATS data collection. After 2020, a new survey replaced both the N-SSATS, and a mental health facility study called the National Mental Health Services Survey [37]. The new survey is called the National Substance Use and Mental Health Services Survey which merges data collected from substance use and mental health treatment facilities [38]. Despite these limitations, this study adds important information concerning the current provision of services available to improve access to SUD treatment.

## Conclusions

Overall, findings from this study highlight both potential facilitators to entering SUD treatment and other barriers being unaddressed based on these four distinct clusters. While clusters of facilities were identified as providing services that serve as facilitators of treatment access, the cluster with the highest proportion of facilities (26.6%) had the fewest services. Further, prominent differences in cluster groupings were identified at the state level. More should be done to ensure that individuals needing SUD treatment are able to access these services.
